# Stretchability and Melt Strength Enhancement of Biodegradable Polymer Blends for Packaging Solutions

**DOI:** 10.3390/molecules30153211

**Published:** 2025-07-31

**Authors:** Katy D. Laevsky, Achiad Zilberfarb, Amos Ophir, Ana L. Dotan

**Affiliations:** Department of Polymer Materials Engineering, Shenkar College of Engineering, Design and Art, Anna Frank 12, Ramat-Gan 6262528, Israel

**Keywords:** packaging, stretchable films, reactive extrusion, biodegradable compounds, compatibilization

## Abstract

Biodegradable polymers offer environmental advantages compared to fossil-based alternatives, but they currently lack the stretchability required for demanding applications such as mesh fabrics for woven flexible intermediate bulk container (FIBC) bags and stretch, shrink, and cling films. The goal of this research is to enhance the stretchability of biodegradable blends based on 80% poly(butylene adipate-co-terephthalate) (PBAT) and 20% poly(lactic acid) (PLA) through reactive extrusion. Radical initiator (dicumyl peroxide (DCP)) and chain extenders (maleic anhydride (MA), glycidyl methacrylate (GMA)) were employed to improve the melt strength and elasticity of the extruded films. The reactive blends were initially prepared using a batch mixer and subsequently compounded in a twin-screw extruder. Films were produced via cast extrusion. 0.1% wt. DCP led to a 200% increase in elongation at break and a 44% improvement in tensile strength. Differential scanning calorimetry and scanning electron microscopy revealed enhanced miscibility between components. Shear and complex viscosity increased by 38% and 85%, compared to the neat blend, respectively. Reactive extrusion led to a better dispersion and distribution of the phases. An improved interfacial adhesion between the phases, in addition to higher molecular weight, led to enhanced melt strength and improved stretchability.

## 1. Introduction

In 2022, 158 million tonnes of plastics entered the packaging sector, effectively being the largest application of plastic by volume [1]. Furthermore, these products tend to have the shortest useful life compared to other industrial plastic applications, emerging as the main source of plastic waste [2], 85% of which ends up in landfills [3].

Polyolefins, particularly low-density polyethylene (LDPE) and polypropylene (PP), are commonly used as packaging materials due to their mechanical properties, low cost, and facile processability. Recycling and reuse are also costly and complex, largely due to the difficulties in separating and properly disposing of mixed plastic waste. Biodegradable plastic alternatives address these challenges by eliminating the need for separation and offering a more sustainable end-of-life solution. To be truly environmentally beneficial, such polymers must not only be compostable but also capable of degrading in soil, where microorganisms break them down into biomass, water, and carbon dioxide. PLA (poly(lactic acid)) and PBAT (poly(butylene adipate-co-terephthalate)) are both biodegradable polymers, but they differ significantly in mechanical properties and degradation behavior. PLA is a strong, stiff material with high tensile strength and modulus [4], making it suitable for rigid packaging. However, it is brittle with very low elongation at break, which limits its use in flexible applications. In contrast, PBAT is a soft, ductile polymer with excellent flexibility and high elongation [5], making it ideal for films and stretchable products, though it has lower strength compared to PLA.

Regarding biodegradation, both polymers are fully compostable under industrial composting conditions [6]. However, their performance in natural environments like soil differs: PBAT degrades more readily due to its aliphatic content [7], while PLA shows very slow or negligible degradation under ambient conditions [8].

Both PLA and PBAT lack stretchability compared to polyolefins. Stretchability refers to a material’s capacity to undergo deformation under tensile stress without breaking, reflecting the balance between its mechanical strength and elongation ability. Stretch properties are important in films because they determine the ability of the film to deform under stress without breaking and the ability to produce flexible and strong meshes for woven FIBC bags and stretch, shrink, and cling films [6]. Several techniques can be applied to improve elasticity in biodegradable polymers, such as filler incorporation [9,10,11] and plasticizer addition [12,13,14], yet these methods are not always capable of providing a satisfactory effect on the mechanical properties. In addition, plasticizers tend to reduce mechanical strength [12] and undergo migration to the surface, making them unstable.

Utilizing reactive extrusion for the compatibilization of biodegradable polymer blends can improve mechanical properties through an increase in molecular weight, chain extension, and potential crosslinking. Chain extension can occur through ring-opening reactions by reacting di- or multi-functional molecules like anhydride, epoxy, isocyanate, and so on with biodegradable polyester’s hydroxyl or carboxylic acid end groups [15]. These methods create mostly biodegradable bonds and, hence, do not substantially affect the degradability [16]. Wang et al. [17] performed reactive melt blending of PLA/PBAT blends with an epoxy-based chain extender Joncryl^®^ ADR 4370S, which enhanced the two-phase adhesion by improving the dispersion at the PLA/PBAT interface. In Andrzejewski et al.’s [18] work, the addition of 0.5 phr of an epoxy-functionalized styrene-acrylic chain extender exceeded the impact strength to 700 J/m due to the growth in branching and entanglements between polymer chains of PLA and PBAT, increasing energy absorption through deformation. Crosslinking is an additional mechanism for compatibilization, which occurs via a free-radical reaction when peroxide is added during the extrusion of the biodegradable blend [19]. This mechanism was studied extensively; Ma et al. [20] investigated reactive blending of PLA with PBAT with dicumyl peroxide (DCP) (0–1 wt.%), which, upon higher DCP content, increased complex viscosity due to the occurrence of branched and crosslinked structures, and the storage modulus, indicating improved elasticity. Enhanced compatibility was evidenced by reduced PBAT domain size and improved interfacial adhesion, as well as increased toughness from 60 J/m to 110 J/m at 0.5 wt.% DCP. The PBAT-g-PLA copolymer formed through the blending process with DCP significantly boosted tensile strength by 72% and elongation at break by 48% in Wu et al.’s [21] study. Despite the benefits, this reaction is difficult to control and could lead to heterogeneity of the final material due to the chain scission reaction. In addition, crosslinks may reduce biodegradation rates [22].

By combining several reactive extrusion methods, this study’s objective is to perform compatibilization of a PBAT/PLA blend rich in the PBAT phase by producing grafted copolymer structures that function as chain extenders themselves, enhancing the melt strength and stretchability of the extruded films, making them suitable for applications that require both flexibility and durability. These polymers, which are inherently incompatible and separate into droplet-like structures of a two-phase system [4], were chosen as their blends are the closest ones to the properties of the polyolefins needed. Their combination can yield a comprehensive performance because of the PLA’s toughness (strength up to 54.7 MPa and a tensile modulus of 3400 MPa) [23] and PBAT’s ductility (elongation at break of 508%) [4]. Radical initiator–chain extender systems were investigated in PLA-rich blends only [24,25], and yet dual-chain extension mechanisms and the effect of residence time have remained unstudied, thus highlighting the novelty of this research.

## 2. Materials and Methods

### 2.1. Materials

PLA, Ingeo biopolymer^TM^ D2003 was obtained from Natureworks LLC. (Blair, NE, USA). It has a melt flow ratio (MFR) of 6 g/10 min (210 °C/2.16 kg). PBAT, Ecoflex^TM^ F Blend C1200, with a melt volume ratio (MVR) of 2.9 cm^3^/10 min (190 °C/2.16 kg), was supplied by BASF SE (Ludwigshafen, Germany). Dicumyl peroxide (DCP) was supplied by Aldrich (Delhi, India), glycidyl methacrylate (GMA) was provided by Sigma-Aldrich (India), and maleic anhydride (MA) was supplied by Fluka Analytical (Cape Town, South Africa).

### 2.2. In-Situ Blend Preparation

#### 2.2.1. Determination of Optimal DCP Content

**Figure 1:**
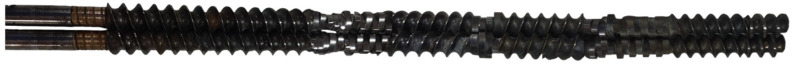
Screw configuration in “Prism Eurolab Digital 16” extruder.

Reactive compounding was performed in a twin-screw extruder “Prism Eurolab Digital 16” with an L/D ratio of 24, a diameter of 16 mm (Figure 1), and a profile temperature range between 150 and 170 °C at 250 rpm for the following blends: 20 wt.% of PLA and 80 wt.% of PBAT with 0.05, 0.1, 0.25, and 0.5 wt.% of DCP.

The obtained pellets were dried at 80 °C in a vacuum oven for 10 h before cast extrusion.

Cast extrusion was performed in a one-screw extruder “Randcastle Extrusion Systems, Inc., Cedar Grove, NJ, USA” with an L/D ratio of 20, a diameter of 13 mm, and a profile temperature range between 150 and 170 °C at 30 rpm, where 300 µm mono-layer films were extruded.

#### 2.2.2. Incorporation of Chain Extenders

After reaching the optimal concentration of DCP (0.1 wt.%), compounds of PBAT/PLA (80/20) with GMA, MA, and DCP were prepared. The chain extenders were added according to a molar ratio to DCP (0.1 wt.%) as depicted in Table 1:

Then, it was mixed with PLA pellets with the help of a stirrer, following dry blending with PBAT pellets. After rheological characterization, those compositions were blended in a batch mixer of “Thermo HAAKE Rheomix OS” (“Thermo Fisher Scientific”, Waltham, MA, USA) at 160 °C and 70 rpm with 10 min of mixing time. The compositions shown in Table 2 had rheological supremacy and were chosen for the reactive extrusion process.

In situ compounding and cast extrusion were executed in the same manner as described above. Optimization of residence time was performed in the DCP–GMA 1:2 blend at a screw speed of 80 and 150 rpm.

### 2.3. Methods

#### 2.3.1. Fourier-Transform Infrared Spectroscopy (FT-IR)

FTIR was performed on 300 µm films of PLA, PBAT, the PBAT/PLA (80/20) neat blend, the PBAT/PLA (80/20) blend with DCP, and PBAT/PLA with DCP and chain extenders, with an ALPHA I FT-IR spectrometer (“Bruker”, Ettlingen, Germany). The spectra were obtained by co-adding 32 consecutive scans within the range of 400–4000 cm^−1^, and the spectral resolution was 4 cm^−1^.

#### 2.3.2. Rheological Behavior

The rheological behavior of the 300 µm reactive extruded films was analyzed via a rotational rheometer with parallel plate-measuring systems of “Discovery HR-1” (“TA Instruments”, New Castle, DE, USA) at 180 °C, in the following methods: flow sweep at a shear rate of 0.1–100 1/s and frequency sweep at a frequency range of 0.1–100 Hz and at 0.5% strain.

#### 2.3.3. Scanning Electron Microscopy (SEM)

SEM “JSM-IT200” (“Jeol”, Tokyo, Japan) was used to study the morphology of the cryogenic fracture of 300 µm reactive extruded films. Film strips were immersed in acetone for 8 h to etch the PLA and expose the morphology, as proposed by the work of Galloway and Macosko [26]. The surfaces were coated with a thin gold layer before observation.

#### 2.3.4. Dynamical Mechanical Analysis (DMA)

“DMA 800” (“TA Instruments”, New Castle, DE, USA) was used to study the visco-elastic properties of the 300 µm reactive extruded films. The tests were performed in dual-cantilever mode, while the storage and loss modulus were recorded as a function of temperature. The Tan(δ) peak temperature refers to the glass transition temperature (T_g_).

The specimens were measured from −50 °C to 95 °C with a temperature sweep of 5 °C/min and at a constant frequency of 1 Hz.

#### 2.3.5. Differential Scanning Calorimetry (DSC)

Thermal analysis via differential scanning calorimetry (DSC) was conducted with “DSC Q250” (“TA Instruments”, New Castle, DE, USA) under a nitrogen atmosphere. Samples of 5–10 mg of 300 µm reactive extruded films were subjected to a heat–cool–heat cycle in the following manner: heating from −85 °C to 200 °C at a rate of 20 °C/min and held for 5 min to reset thermal history. Subsequently, they were cooled to −85 °C at the rate of 20 °C/min to obtain the melt crystallization temperature (T_c_) and corresponding crystallization enthalpy (ΔH_c_), followed by heating to 200 °C at a rate of 20 °C/min to obtain the glass transition temperature (T_g_), melting temperature (T_m_), and melt enthalpy (ΔH_m_). The degree of crystallinity was calculated with the following equation: $Xc=\frac{\left(\Delta Hm-\Delta Hcc\right)}{\Delta {Hm}^{0}\cdot w}\cdot 100$, where ΔH_m_^0^ PLA = 93.7 J/g [21], ΔH_m_^0^ PBAT = 114 J/g [21], and w is the weight fraction of the polymer in the blend.

#### 2.3.6. Tensile Testing

A tensiometer, “Instron 4481” (“Instron”, Norwood, MA, USA) was used to study the tensile properties of 300 µm reactive extruded films. Films were cut into rectangular-shaped strips according to ASTM D882. The crosshead speed used was 500 mm/min. Ten film specimens were tested for each composition ratio.

## 3. Results and Discussion

### 3.1. Determination of Optimal DCP Content

DCP incorporation in PBAT/PLA blends promotes partial crosslinking reactions due to the formation of macro-free radicals because of the hydrogen abstraction that occurs during radical reactions. PBAT has a higher capacity to undergo hydrogen abstraction due to the high content of mobile hydrogen atoms compared to PLA [27].

Due to the difficulty in controlling the kinetics of the radical reaction, different structures are formed: partially crosslinked PLA and PBAT, networks of PLA-crosslinked-PBAT, and copolymers of PLA-g-PBAT [20], which are formed by the radical coupling mechanism.

Chain scission may occur depending on the amount of radical initiator integrated [28].

**Figure 2:**
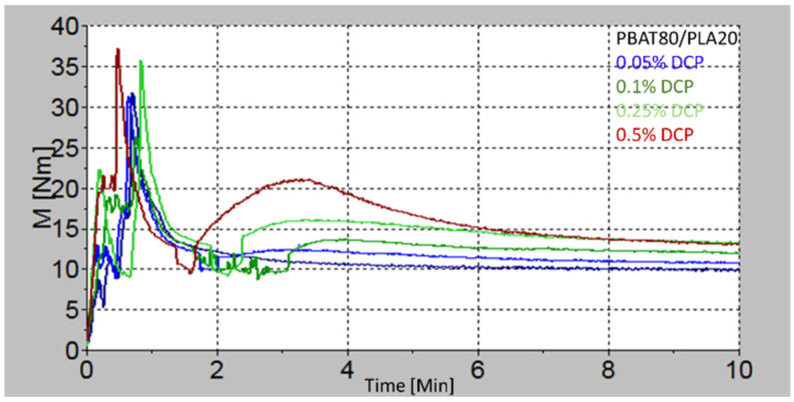
Torque measurement during batch-mixing of PBAT/PLA (80/20) with changing DCP content.

Screw torque was measured during the batch-mixing process (Figure 2). DCP addition doubled the torque values in the 0.5% DCP blend compared to the neat blend, establishing the crosslinking/branching reaction.

#### 3.1.1. FT-IR Analysis

**Figure 3:**
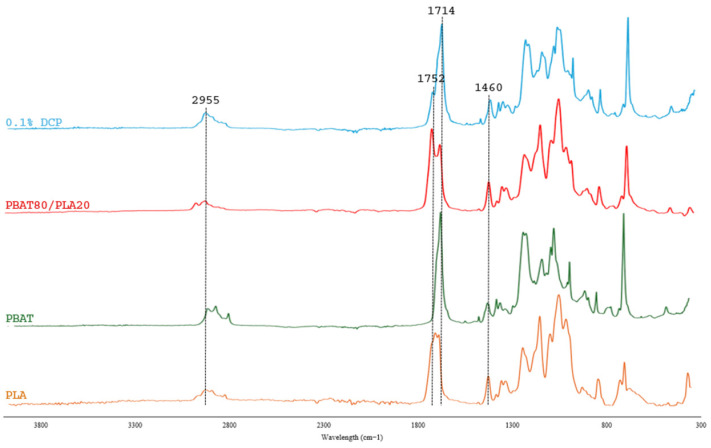
FT−IR spectra of pure PLA, pure PBAT, PBAT/PLA (80/20) blend, and blend with 0.1% DCP.

To assess the structural changes and interactions between the PBAT and the PLA polymers due to reactive blending, FT-IR spectra were characterized in Figure 3.

In PBAT’s spectra, the absorbance bands at 2849, 2918, and 2958 cm^−1^ correspond to the elastic vibration of C–H [29]. These peaks were weaker in the PLA’s spectra due to fewer aliphatic –CH_2_– groups. Additionally, bending of the aromatic C–H in PBAT adds peaks in the fingerprint region (900–700 cm^−1^) [30]. Both polymers exhibited an absorbance peak at 1460 cm^−1,^ which was caused by the flexural vibration of –CH_3_ [29].

For the PBAT/PLA spectra, absorbance peaks at 1714 cm^−1^ and 1752 cm^−1^, indicate –C=O stretching vibrations originating from the ester groups, implying interactions between the polymers [21]. A sharp peak at 720 cm^−1^ was observed in PBAT, PBAT/PLA, and 0.1% DCP spectra, representing –CH_2_– adjacent methylene groups [30].

Incorporation of DCP elevated the absorbance intensity of the C–H stretching band at 2955 cm^−1^, which implies the presence of crosslinks and branches since the –CH_2_– groups are being attacked by free radicals and transformed into C–H [31]. A reduction in absorbance intensity was observed at 1752 cm^−1^, indicating the formation of new esters, likely due to chain scission.

#### 3.1.2. Rheological Properties

The viscoelasticity of polymer blends can be defined by the storage modulus (G′) component, which represents the polymer’s elastic property, and the loss modulus (G″), which portrays viscous ability.

**Figure 4:**
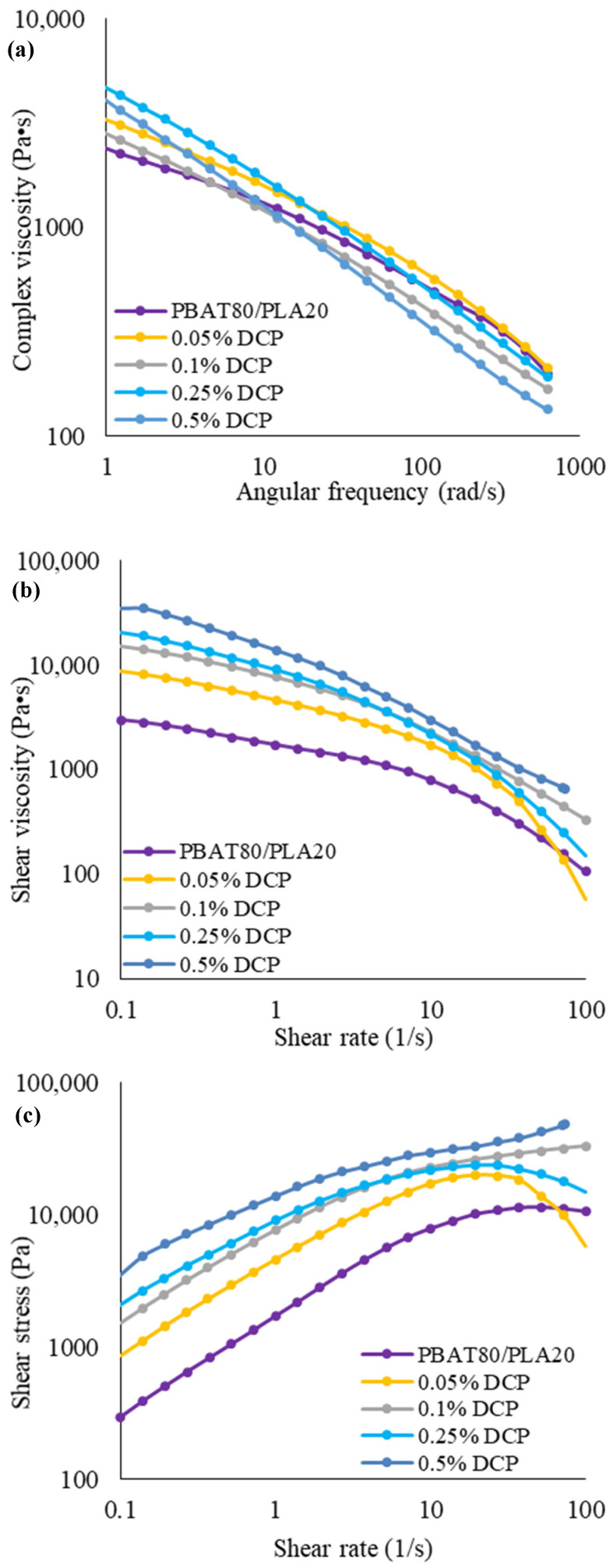
Rheological behavior of PBAT/PLA (80/20) extruded blends as a function of frequency, shear rate, and DCP content: (**a**) complex viscosity, (**b**) shear viscosity, and (**c**) stress.

Both the loss and storage moduli of all the blends tested increased over the entire frequency range, indicating an improvement in those properties. Shear-thinning behavior is observed in Figure 4a, with it becoming stronger with the increasing shear rate and angular frequency. At low angular frequencies (ω < 4.52 rad/s), all the blends exhibited equal or higher complex viscosity compared to the neat blend. At high angular frequencies (ω > 87.3 rad/s), only the blend with 0.05% DCP displayed higher complex viscosity than the neat blend.

The incorporation of DCP altered the shear sensitivity of the tested blends. The slopes in η(γ) of blends with 0.05% and 0.25% DCP were higher than the neat blend slope, indicating an increase in shear sensitivity. The reduction in shear stress of the 0.05% DCP blend was spotted at shear rates higher than 27 s^−1^, implying shear-induced changes in the blend’s morphology, whereas the smaller slopes of blends with 0.1% and 0.5% DCP are attributed to lower sensitivity due to cohesive network formation [32]. A decrease in the slope of storage modulus (frequency) was demonstrated with the addition of DCP, yet no plateau was displayed due to chain extension [33].

**Figure 5:**
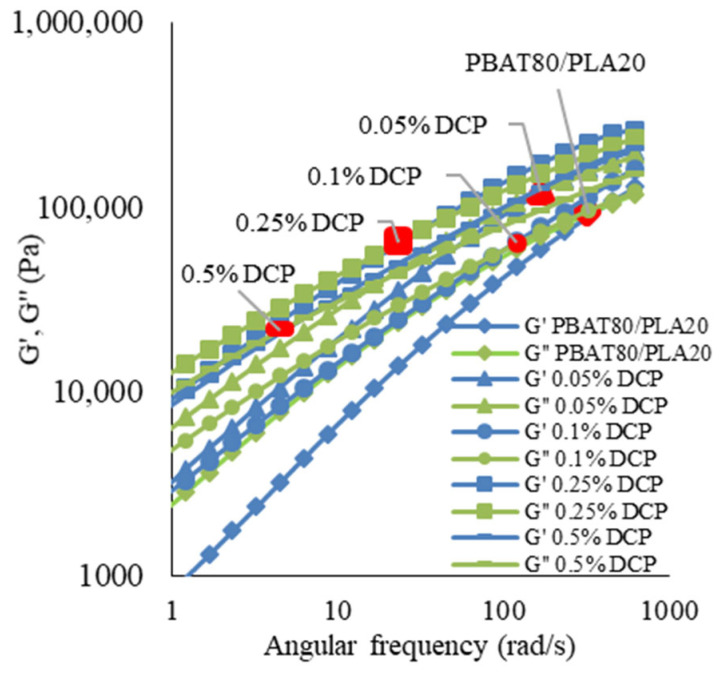
Storage and loss modulus crossover points from frequency sweep curves of PBAT/PLA (80/20) batch-mixed blends as a function of DCP content.

**Figure 6:**
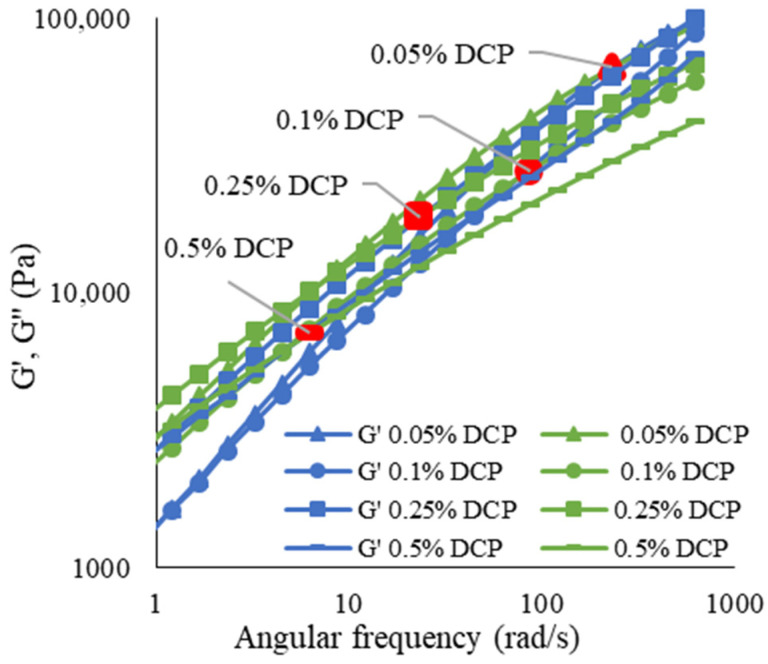
Storage and loss modulus crossover points from frequency sweep curves of PBAT/PLA (80/20) extruded blends as a function of DCP content.

The value of the angular frequency at the intersection between G′ and G″ curves reflects the degree of entanglement of molecular chains in polymer melts with physical cross-linking (Table 3). A higher degree of entanglements led to a smaller value of ω at the intersection point [34]. A high amount of DCP lowered the ω at the intersection point, as depicted in Figure 5 and Figure 6, indicating higher entanglements of the molecular chains in the polymer blends that elevate melt strength and shear viscosity. In addition, the crossover point of G′, G″ shifted to lower modulus and frequency values, indicating growth in molecular weight (Mw) and broadening of the molecular weight distribution (MWD) [35], which confirms chain scission due to a stronger radical reaction as a result of greater DCP concentrations.

The transition from batch mixer to extrusion reduced the modulus of the intersected G′, G″(ω) point in all blends. This indicates broadening of the MWD, which sheds light on the existing shear regime in the extrusion process and its influence on the chain scission reaction.

Blends with 0.5% DCP displayed the highest shear viscosity in shear rates tested, yet exhibited the biggest slope in complex viscosity during oscillatory stress. This behavior could be explained by the increased flow resistance of highly branched polymer chains, which elevates shear viscosity [36], with the self-lubrication phenomena that the shorter polymer chains account for [37]. These chains, which broaden the MWD, allow some disentanglement of the cross-linked network, thus leading to a decrease in viscosity at higher frequencies [38].

**Figure 7:**
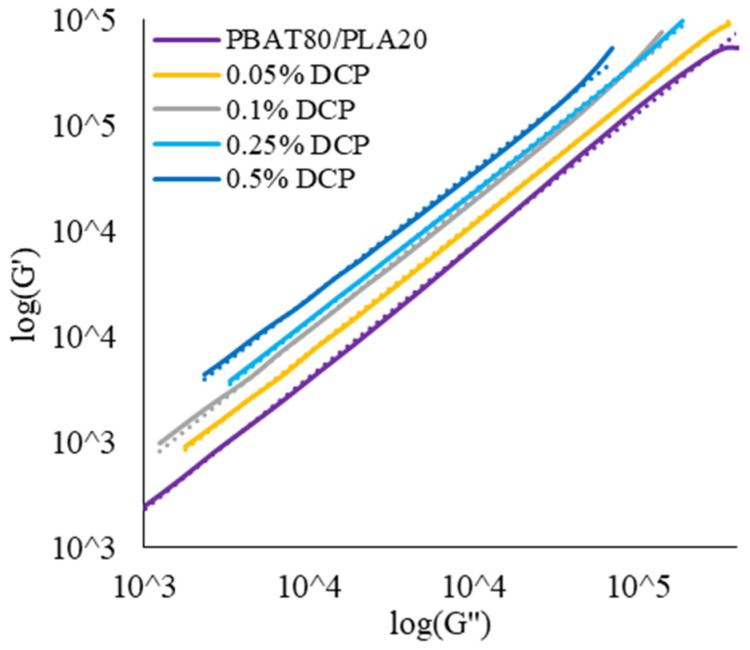
Han plot for the PBAT/PLA (80/20) blends as a function of DCP content.

The Han plot (Log G′ versus log G″) sheds light on blend compatibility and phase behavior. The straight line of log G′ = log G″ indicates the transition from elastic (G′ > G″) to viscous (G′ < G″) behavior. A higher amount of DCP distanced the lines further from the log G′ = log G″ to the elastic region, as seen in Figure 7 and Table 4, indicating a more dominant elastic property and improved compatibility [39]. This observation fits in with the increment in elongation, as observed in the tensile test. In addition, improved elasticity occurs due to increased branching reactions with a high degree of entanglements in the formed network [40].

**Figure 8:**
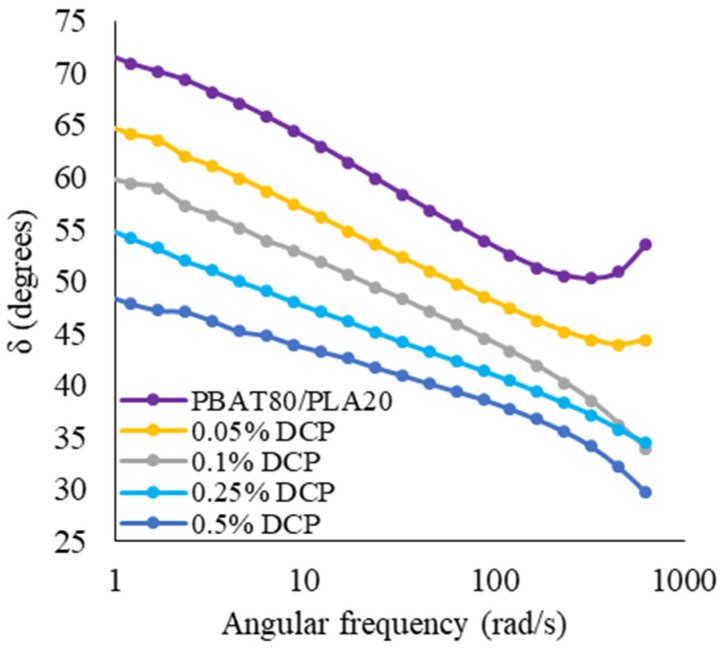
Loss angle versus angular frequency of PBAT/PLA (80/20) blends as a function of DCP content.

The loss angle (δ) that is calculated from tan δ = G″/G′ sheds light on the material’s ability to dissipate energy. As shown in Figure 8, the loss angle increases with decreasing angular frequency. Phase angles diminished with the growing amount of DCP incorporated in the blend, indicating improvement in melt elasticity—chain-scissioned segments act as plasticizers.

The neat blend exhibited the highest phase angles in the tested frequency range. The blend’s immiscibility, which accounts for a two-phased droplet system, establishes its potency to dissipate energy under applied shear stress.

#### 3.1.3. Thermal Analysis

The thermogram of the PBAT displayed two melting and crystallization points, indicating a bimodal compound. PLA exhibited a cold crystallization peak at 131 °C. Two melting transitions were detected in blend thermograms, implying the formation of a two-phase system due to the incompatibility between PLA and PBAT polymers.

Figure S1 displays the calculated shift in T_g_ values of both phases from the second heating cycle in Table 5.

**Figure 9:**
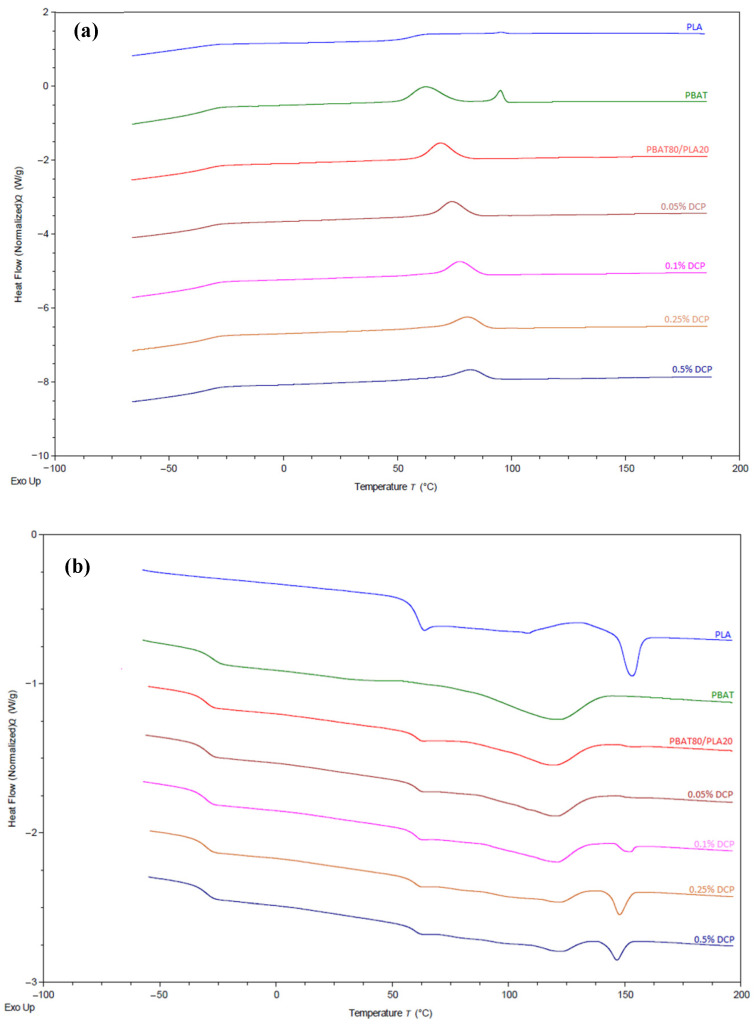
Thermograms of the (**a**) first cooling curves and the (**b**) second heating curves of pure PLA, pure PBAT, neat blend, and blends with different DCP content.

Blends with 0.05%, 0.1%, and 0.25% DCP displayed inward shifts of the PLA and PBAT glass transition peaks, as seen in Figure 9, implying improved blend miscibility and thus better interfacial adhesion between the consistent polymers. An outward shift was exhibited in the 0.5% DCP blend, indicating inferior compatibility.

Higher DCP content elevated the crystallization temperature (T_c_) due to crosslinking, which improves the melt crystallization of PBAT [41], yet reduces the heat crystallization (ΔH_c_) during cooling. PBAT’s higher potency forming macroradicals compared to PLA segments leads to more PBAT segments that are branched and entangled, which suffer from restricted movement that impedes their ability to create a crystalline lattice, which is manifested in the reduction in PBAT’s X_c_ with the growing wt.% of DCP.

The degree of crystallization for PLA in the blends cannot be reliably calculated due to the overlap of PLA’s cold crystallization and PBAT’s melting range, which is in agreement with the observation of Su et al. [42].

An elevation in melting temperature (T_m_) of the PBAT phase was exhibited, in addition to a reduction in heat melting (ΔH_m_). The decrement observed in heat melting of the PBAT phase and heat crystallization indicates a lower degree of crystallization (X_c_) [43], as demonstrated in Table 5. In addition, the rise in heat melting (ΔH_m_) with the reduction in the melting temperature (T_m_) of the PLA phase may be due to interface formation [44].

#### 3.1.4. Morphological Study

**Figure 10:**
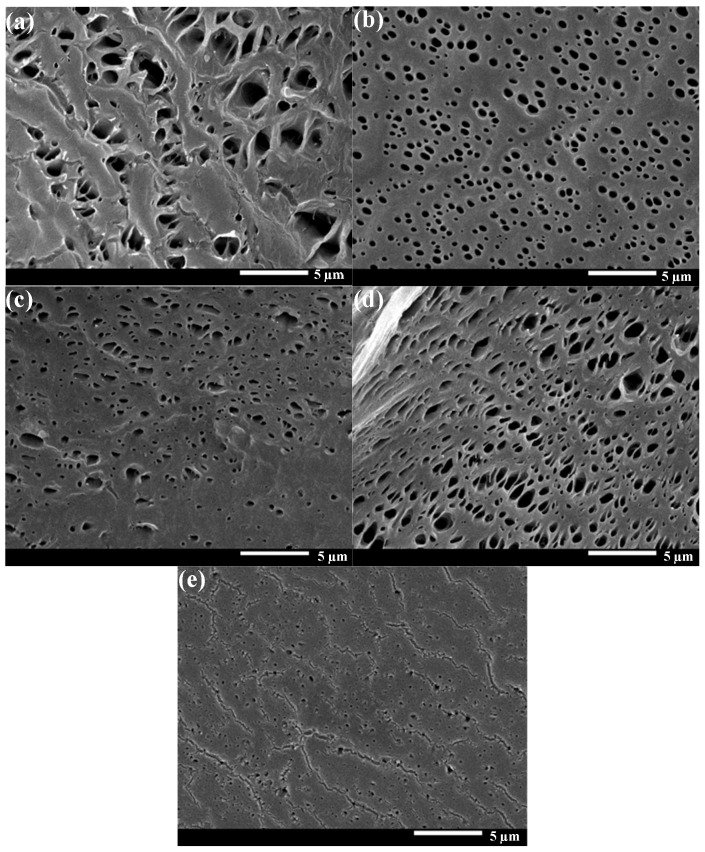
Morphology of PBAT/PLA (80/20) blends with DCP: (**a**) neat blend, (**b**) 0.05%, (**c**) 0.1%, (**d**) 0.25%, and (**e**) 0.5%; obtained by SEM, ×5000.

After PLA etching, all blends exhibit pore-like structure remains where droplets of PLA were dispersed, indicating that interface de-bonding is the dominating fracture micro-mechanism [45]. In Figure 10a, the fibrils that connect the PBAT phase with the pore residues indicate a ductile fraction due to the PBAT ductile features. The first signs of compatibilization can be observed in Figure 10b, where boundaries of the dispersed phase have a more defined spherical manner. Enhanced compatibilization occurs when the size of the dispersed phase diminishes with the growing particle size distribution due to a reduction in interfacial tension that stabilizes the dispersed phase [25], as seen in Figure 10c. Figure 10d presents a high dispersion of different-sized distributed pores, and STDEV.S supports this observation (Table 6).

The SEM image shows that the dispersion and the size of the dispersed phase change with an increase in DCP content. Higher DCP content (0.5%) features smaller pores and more uniform distribution and dispersion of the PLA particles, which agrees with the lowest average particle size and STDEV.S.

#### 3.1.5. Tensile Properties

The inclusion of PBAT in the PLA matrix grants ductility to the PBAT/PLA blend due to a synergistic effect [4].

**Figure 11:**
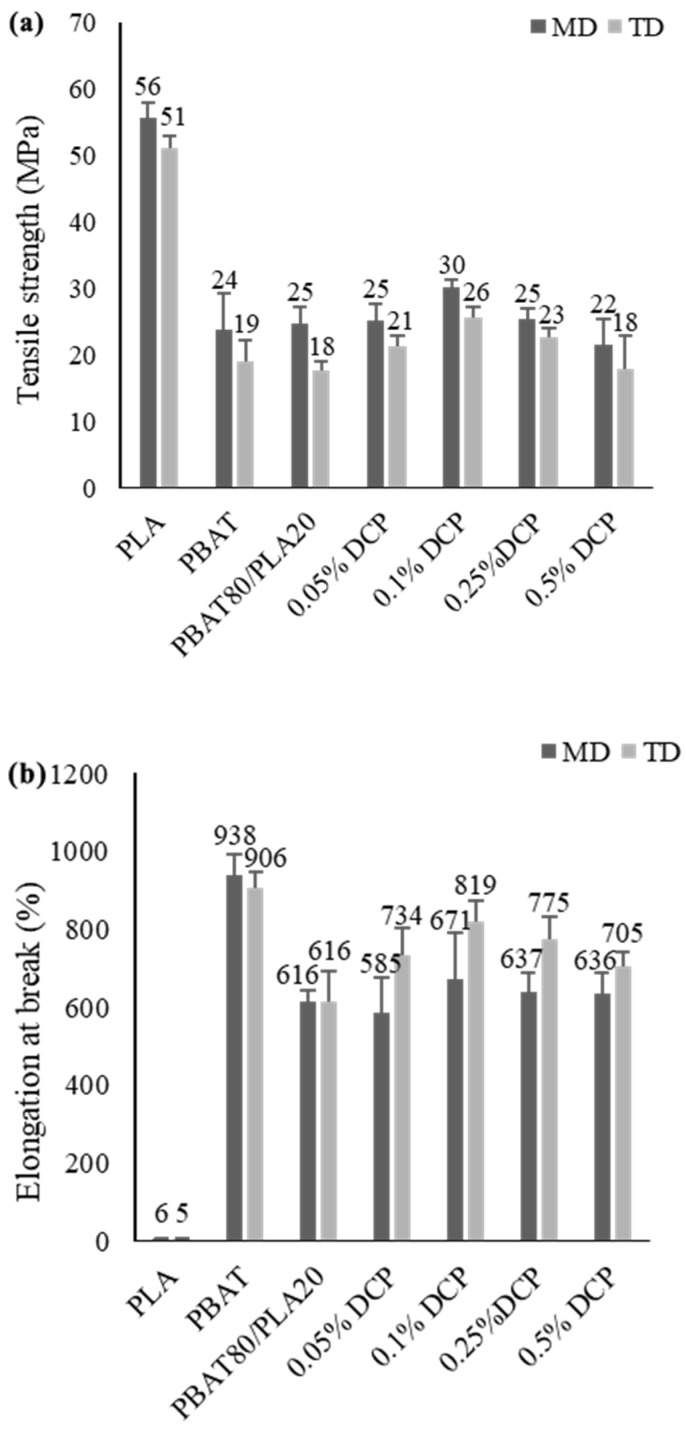
Tensile properties of PBAT/PLA (80/20) blends as a function of DCP content: (**a**) tensile strength, (**b**) elongation at break.

As seen in Figure 11, the PBAT/PLA blend with 0.1% DCP exhibited the greatest enhancement in tensile strength and elongation at break, both in the machine direction (MD) and transverse direction (TD); tensile strength improved by 20% in MD and 44% in TD, while elongation at break was elevated by 55% in MD and 203% in TD. The improved miscibility validated by DSC analysis, along with the reduction in the dispersed phase as was manifested in SEM, all point to the improved stretchability of the specific blend [20,21]. Additional information from the tensile test is summarized in Table S1.

The films exhibited higher tensile strength in the machine direction due to specific molecular orientation during film extrusion, whereas molecules align more favorably for elongation in the transverse direction.

An additional increase in DCP content (above 0.1%) caused degradation in the properties mentioned due to chain scission (a 20% decrease in tensile strength in MD in the 0.25% DCP blend, followed by an additional 14% reduction in the 0.5% DCP blend), which promotes shorter polymer chains with fewer entanglements and weaker intermolecular interactions, which have a limited ability to uncoil during applied stress and over-crosslinking, which causes embrittlement [46].

#### 3.1.6. Dynamical-Mechanical Behavior

**Figure 12:**
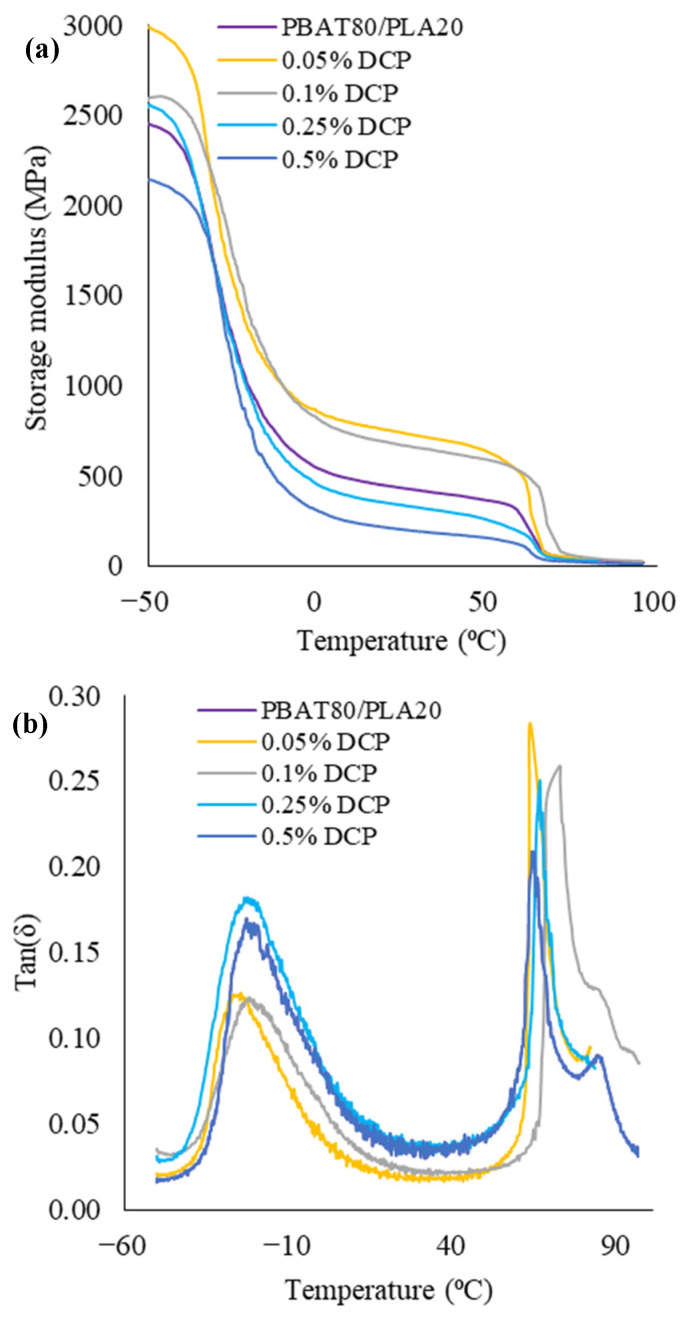
Dynamical-mechanical tests on PBAT/PLA (80/20) blends as a function of DCP content and temperature: (**a**) storage modulus, (**b**) tan (δ).

As seen in Figure 12a and Table S2, each blend exhibited two glass transitions, one to each matrix, which indicates the immiscibility of the components. The addition of DCP affected the T_gs_ of PLA and PBAT phases—the highest values were displayed in the 0.1% DCP blend, where the T_g_ of PBAT and PLA domains increased by 5 °C and 6 °C, respectively, indicating enhanced compatibility [20].

Due to the synergistic effect, the storage modulus of the PBAT/PLA blend is lower than neat PLA in the tested temperature range. Blends with 0.05 and 0.1% DCP exhibited higher storage modulus values than the neat blend in the tested temperature range, indicating greater stiffness due to crosslinking formation, leading to a more rigid network structure. This is manifested in the improved tensile strength, as seen in Figure 11.

Only the blend with 0.25% of DCP displayed an inversion in the storage modulus behavior—at temperatures lower than the T_g_ of PBAT, storage modulus values were higher than the neat blend, while at higher temperatures, where PBAT was rubbery, storage modulus values were lower than the neat blend. This behavior can be explained by the inherent phase separation between PLA and PBAT components, which intensifies in the particular blend when the temperature rises, diminishing interfacial adhesion, and thus reducing the blend’s stiffness.

The blend with 0.5% DCP exhibited the lowest storage modulus values in the temperature range tested, which signifies lower stiffness due to poor compatibilization leading to a deterioration in tensile strength, as is shown in Figure 11, thus supporting the observation of the decreasing size of PLA droplets in the PBAT matrix, as seen in Figure 10e.

Stronger radical reaction occurs with an increasing amount of DCP, which promotes chain extension on the one hand and the chain scission process on the other hand. This effect was confirmed by the decrement in mechanical properties for blends with more than 0.1% DCP, as seen in the tensile test and the dynamical-mechanical analysis.

After concluding the analysis, 0.1% DCP was chosen as the optimal concentration for further reactive extrusion of chain extenders in the examined blend.

### 3.2. Incorporation of Chain Extenders

The grafting reaction is promoted by chain extender addition, such as MA and GMA, to PLA and PBAT polymers [47].

**Scheme 1:**
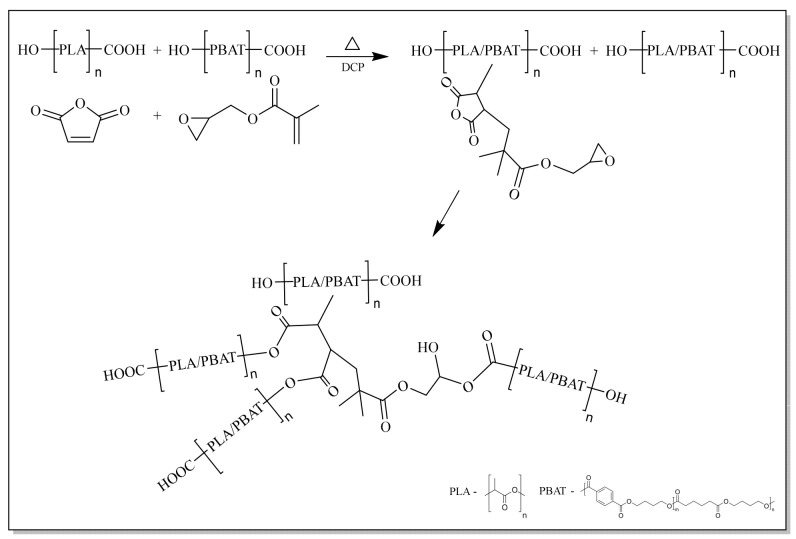
Proposed mechanism for grafting reaction during in situ compatibilization.

As can be seen in Scheme 1, in the presence of DCP, MA reacts through its double bond with the macro-free radicals of PLA and PBAT [24] to form PLA-g-MA and PBAT-g-MA structures. When added, GMA reacts through its tertiary carbon to form PLA-g-GMA and PBAT-g-GMA structures.

The formed grafted structures function as chain extenders themselves by ring-opening reactions in the following manner: epoxide on the GMA group reacts with the terminal carboxyl groups on PLA and PBAT, while the cyclic structure on the MA group reacts with the terminal hydroxyl groups [48].

The abovementioned grafted structures account for physical entanglements with PLA and the PBAT matrix [25].

#### 3.2.1. FT-IR Analysis

**Figure 13:**
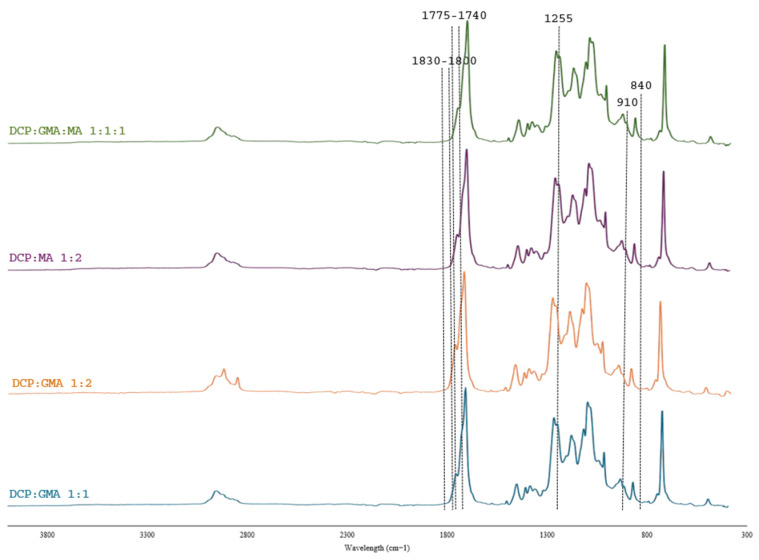
FT-IR spectra of PBAT/PLA (80/20) extruded blends with different GMA, MA, and DCP content.

The introduction of chain extenders to a 0.1% DCP blend have increased the overall intensity in existing functional groups due to multiplication of the bonds as a result of chain extension (C–O stretches at ~1300–1000 cm^−1^, C=O stretching vibrations at ~1750–1710 cm^−1^ and C–H elastic vibration at ~3000–2800 cm^−1^), which can be seen in Figure 13.

The epoxide functional group on the GMA molecule exhibits characteristic absorbance peaks at 840, 910, and 1255 cm^−1^, attributed to the C–O stretching vibration [49]. Since those peaks were not observed in the DCP–GMA 1:2 blend, it can be deduced that all the epoxide rings have reacted with the terminal carboxyl groups of PLA and PBAT during reactive extrusion. This observation was also presented by Wu et al. [21].

Typical absorbance peaks for the anhydride functional group in MA, corresponding to C=O stretching, are within the following ranges: 1775–1740 cm^−1^ and 1830–1800 cm^−1^ [50,51]. However, these peaks were not present in the DCP–MA 1:2 blend, as was also detected by Barbosa et al. [52]. Existing peaks in the 1775–1740 cm^−1^ range are overlapping, and new peaks characterizing MA did not occur due to ring-opening reaction with terminal hydroxyl groups of PLA and PBAT [48].

The combination of these in the DCP–GMA–MA 1:1:1 blend led to similar observations as those described above.

No evidence of the grafted PLA, PBAT-g-GMA, or MA structures was present in the spectra due to the occurrence of the ring-opening reaction during extrusion.

#### 3.2.2. Rheological Properties

**Figure 14:**
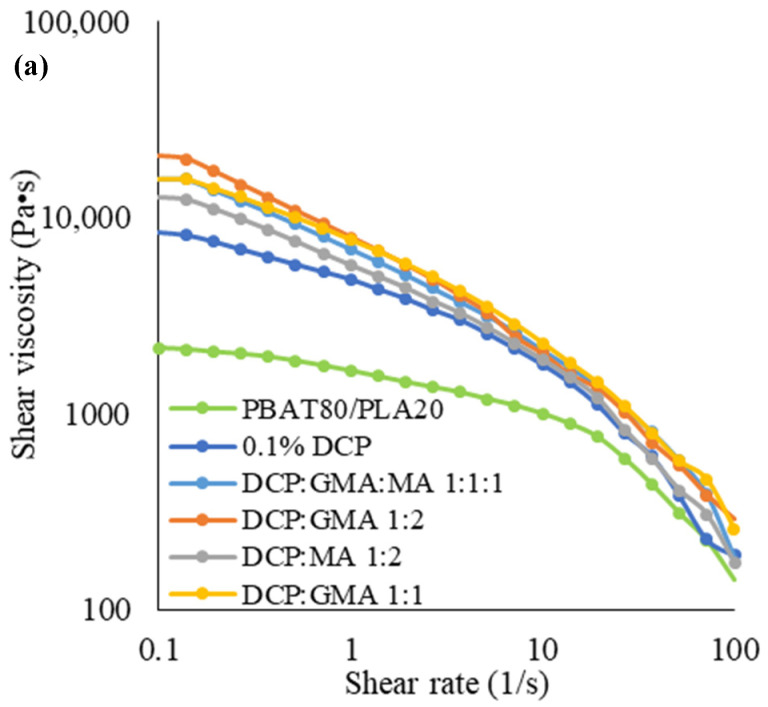

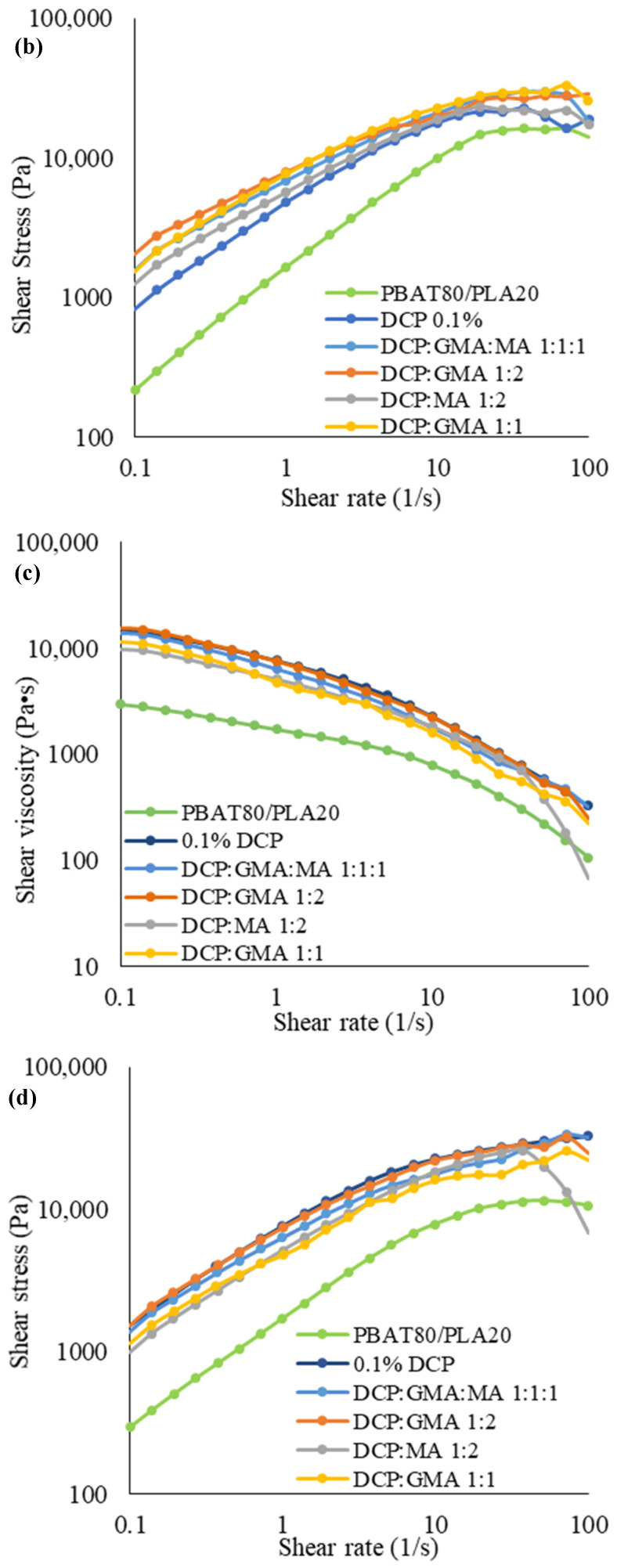
Rheological behavior of PBAT/PLA (80/20) blends as a function of shear rate, DCP, GMA, and MA content: (**a**) shear viscosity of batch-mixed blend, (**b**) stress of batch-mixed blend, (**c**) shear viscosity of extruded blend, and (**d**) stress of extruded blend.

All chain extender–DCP batch-mixed blends exhibited improved shear viscosity and melt strength compared to a batch-mixed blend of 0.1% DCP, as depicted in Figure 14, due to the occurrence of entanglements and chain extension due to the grafting structures.

A difference of 38% was spotted between the shear viscosity of the DCP–GMA 1:1 batch-mixed blend to an extruded blend at a shear rate of 1 s^−1^, 7716 to 4767 Pa•s. This may occur due to the insufficient time for the ring-opening reaction (shorter residence time) in the transition between the batch mixer and extrusion [53].

Inverse behavior was spotted in the 0.1% DCP blend, where the transition from batch mixer to extruder raised melt strength and shear viscosity. This goes hand in hand with the growing Mw that can be observed in Figure 6 and can be explained by the occurrence of chain scission as a result of continuous shear forces.

**Figure 15:**
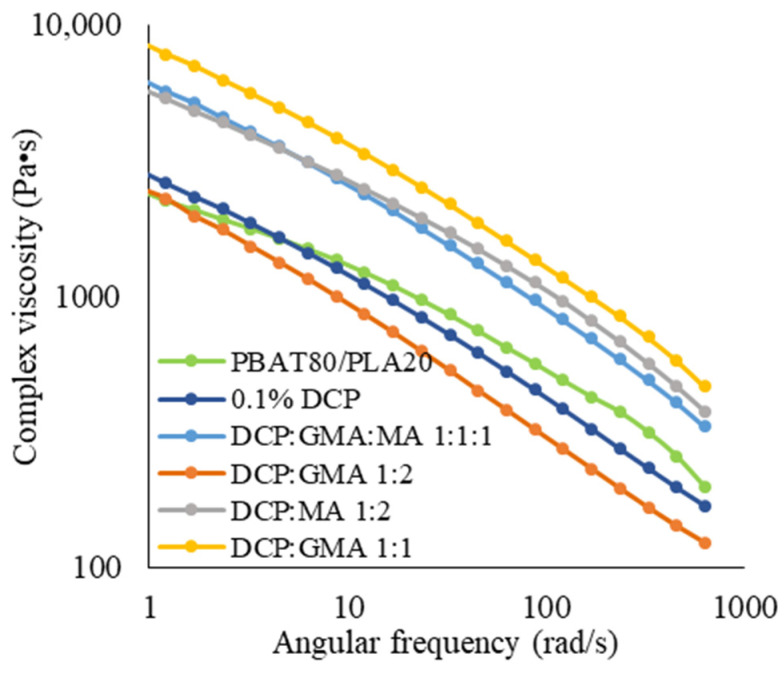
Complex viscosity of PBAT/PLA (80/20) extruded blends.

According to Figure 15, extruded blends with DCP–GMA 1:1, DCP–GMA–MA 1:1:1, and DCP–MA 1:2 displayed higher complex viscosity values than the neat blend, the blend with 0.1% DCP, and the DCP–GMA 1:2 blend.

**Figure 16:**
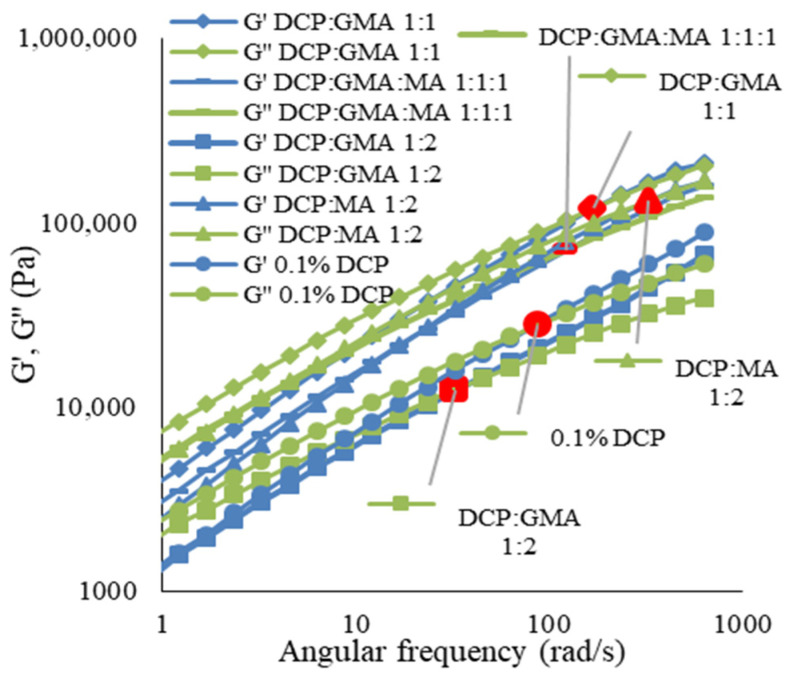
Storage and loss modulus crossover points from frequency sweep curves of PBAT/PLA (80/20) extruded blends as a function of DCP, GMA, and MA content.

**Figure 17:**
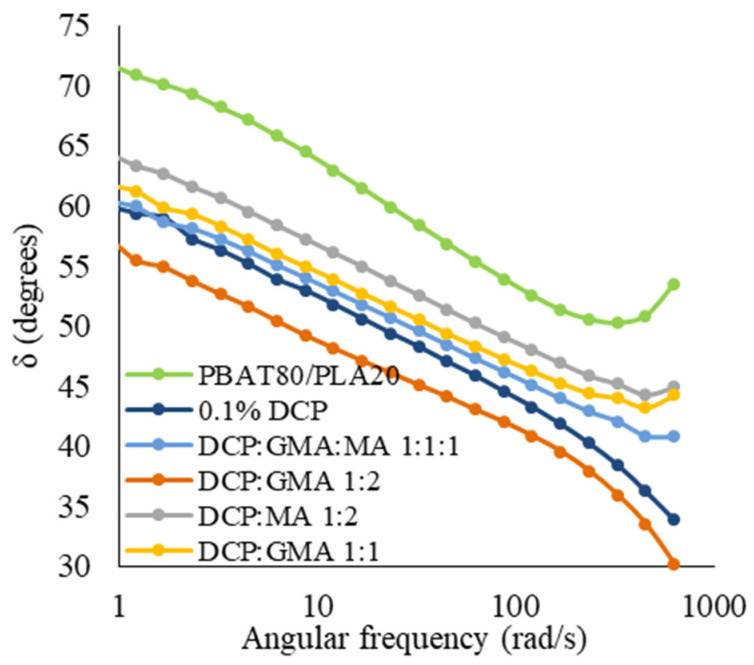
Loss angle versus angular frequency of PBAT/PLA (80/20) extruded blends as a function of DCP, GMA, and MA content.

**Figure 18:**
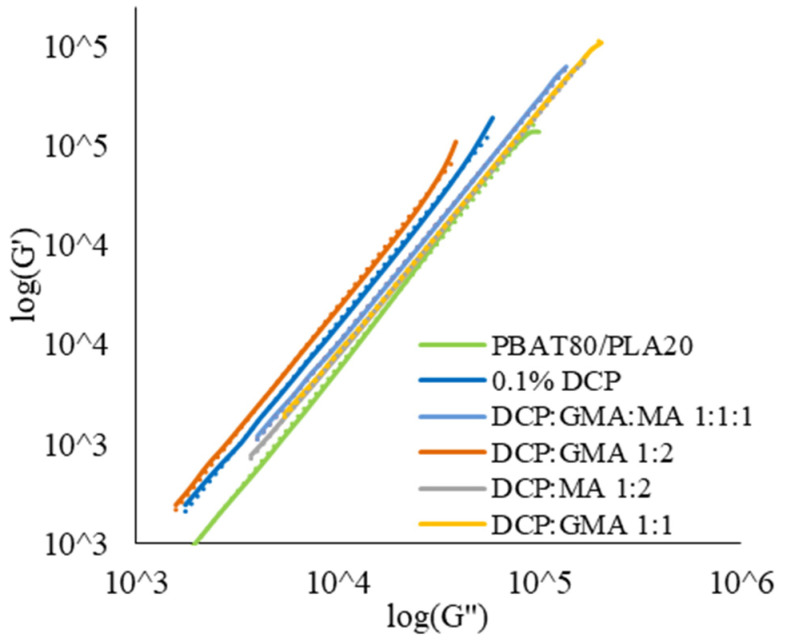
Han plot of PBAT/PLA (80/20) extruded blends as a function of DCP, GMA, and MA content.

According to Figure 16 and Table S3, the blend with DCP–GMA 1:2 displayed the smallest value of ω at the intersection point, indicating the highest degree of entanglement of molecular chains, yet this blend exhibited the lowest complex viscosity value. This phenomenon could be explained by the crossover point G′, G″(ω) of the DCP–GMA 1:2 blend, which points to the highest and broadest MWD. Grafting and crosslinking occurring during the reactive extrusion process create different-sized polymer chains that broaden the MWD in the melt, increasing chain mobility, which diminishes the overall resistance to flow, lowering complex viscosity [38]. This blend also exhibited the lowest phase angles, as seen in Figure 17, indicating the greatest enhancement in melt elasticity, supporting the dominant elastic property seen in the Han plot in Figure 18 and Table 7, which implies that the short polymer chains act as plasticizers.

#### 3.2.3. Thermal Analysis

**Figure 19:**
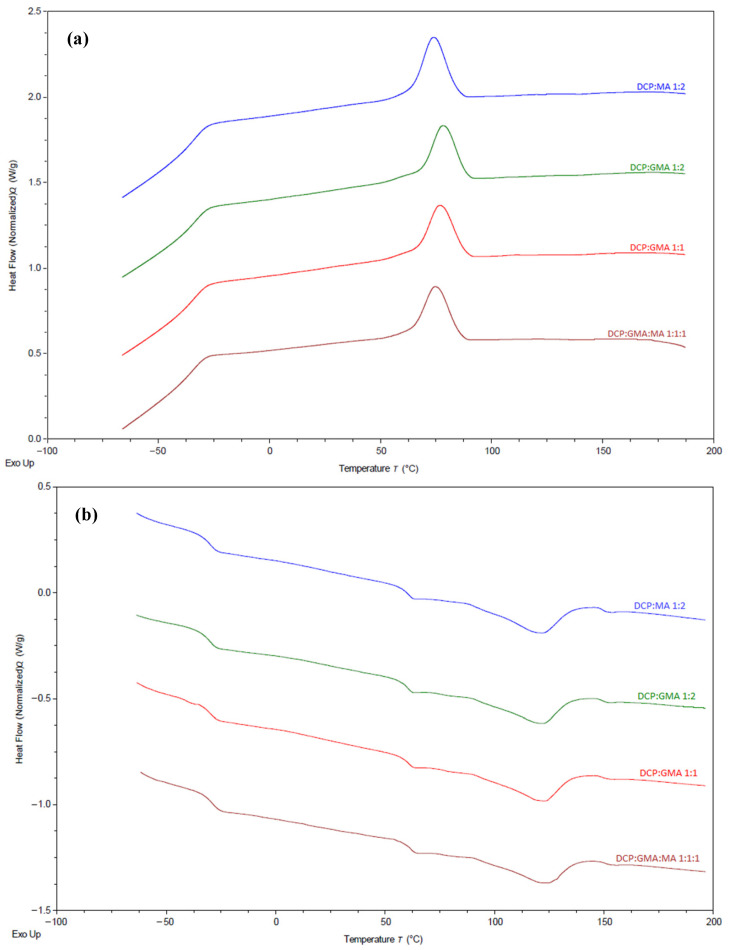
Thermograms of the (**a**) first cooling curves and the (**b**) second heating curves of PBAT/PLA (80/20) extruded blends with different GMA, MA, and DCP content.

The remaining presence of the two glass transitions, seen in Figure 19b, indicates a two-phase system despite the enhancement in melt strength and complex viscosity as proven above.

Disappearance of PLA’s Tm (second cycle) occurred in all blends containing chain extenders along with DCP due to restricted crystallization of the PLA phase as a result of chain extension phenomena [21]. A slight elevation in PBAT’s Tm (second cycle) was exhibited (0.9–2.9 °C) and could be attributed to an increase in lamellar thickness [7].

The incorporation of chain extenders in addition to DCP promoted the formation of grafted structures, which hinder chain mobility. A reduction in heat crystallization and X_c_ of the PBAT phase was spotted during DSC, which can be attributed to PBAT’s higher capability to undergo grafting compared to PLA segments, as a result of its favorability to form macroradicals.

The highest T_c_ was recorded for the blend with DCP–GMA 1:2 (78.2 °C), along with the lowest heat crystallization (ΔH_c_ = 12.1 J/g), which indicates higher nucleation stability of the PBAT phase. This stability is granted due to increased viscosity, which reduces the thermal random motion of polymeric chains [21].

The blend with DCP–GMA 1:1 displayed an inward shift of the T_g_ (second cycle, Figure S3 from Table 8) compared to the shift in the blend with 0.1% DCP, suggesting enhanced thermodynamic miscibility. This corresponds to the highest values of complex viscosity and the narrowest MWD, indicating the formation of more uniform long-chain structures.

#### 3.2.4. Morphological Properties

The introduction of chain extenders increased the dispersed phase size (Table 9) due to the coalescence of adjacent pores [54] in all blends compared to the blend with 0.1% DCP

**Figure 20:**
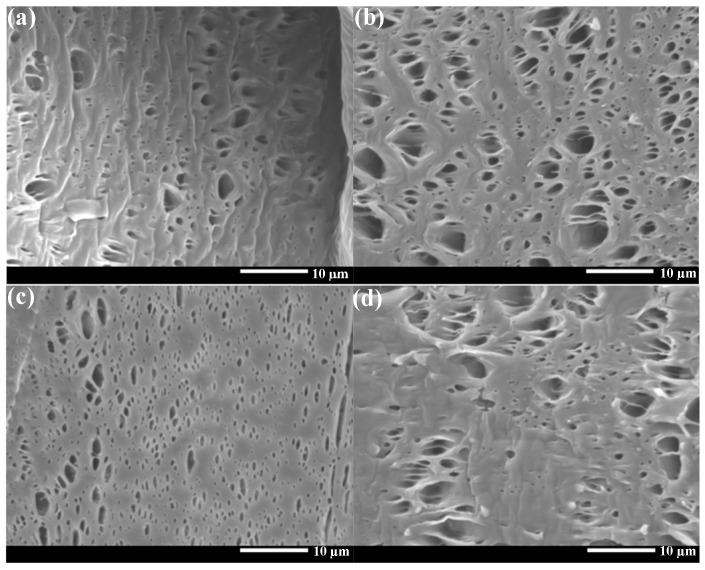
Morphology of PBAT/PLA (80/20) extruded blends with DCP, GMA, and MA: (**a**) DCP–GMA 1:1, (**b**) DCP–GMA–MA 1:1:1, (**c**) DCP–GMA 1:2, (**d**) DCP–MA 1:2, obtained by SEM, ×2500.

Changes in morphology could be detected in blends with different GMA-to-DCP ratios. A higher concentration of GMA (Figure 20c) slightly reduced the dispersed phase size and granted more defined boundaries with a smoother fracture surface, which could be attributed to the reduction in the interfacial tension, as a result of the grafting structures formed at the interface [55]. The ductile fracture was exhibited in the blend with DCP–GMA 1:1.

Blends with DCP–GMA–MA 1:1:1 and DCP–MA 1:2 exhibited similar surface fraction appearance—large cavities, adjacent different-sized pores connected through fibrils, and gliding areas. This heterogeneous morphology indicates further phase separation and is supported by the outward shift of T_g_ seen in the thermal analysis, indicating thermodynamic immiscibility.

#### 3.2.5. Tensile Properties

All compatibilized blends displayed enhanced mechanical properties compared to the neat blend.

Slight improvement in elongation at break in MD was presented in blends with DCP–GMA–MA 1:1:1 and DCP–MA 1:2 compared to 0.1% DCP (20% and 15%, respectively), which can be attributed to the heterogeneous morphology that plasticized the blends [16].

**Figure 21:**
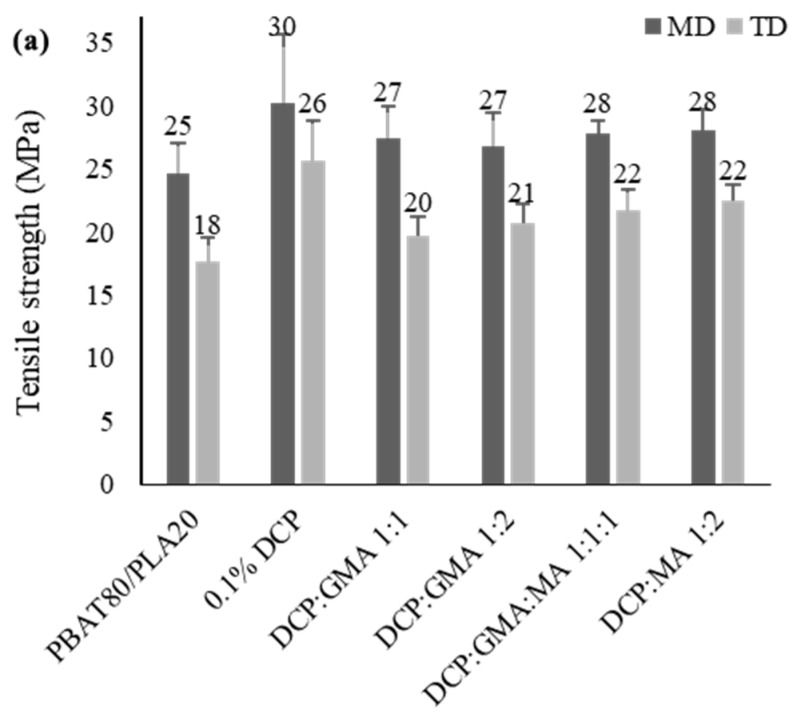

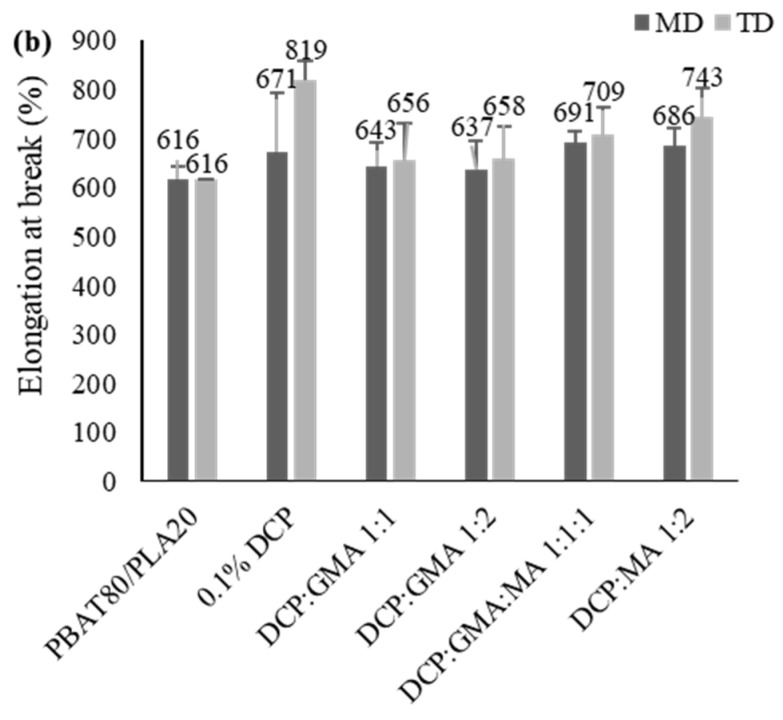
Tensile properties of PBAT/PLA (80/20) extruded blends with DCP, GMA, and MA: (**a**) tensile strength, (**b**) elongation at break.

As seen in Figure 21 and Table S4, the addition of chain extenders in the current set of process parameters did not improve mechanical properties substantially due to partial ring-opening reactions of the grafted chain extenders as a result of insufficient residence time in the extruder [53]. This corresponds with the deterioration in rheological properties discussed above.

Additionally, side reaction between chain extenders and free radicals, originating from the DCP [20], hinders radical reactions between polymeric chains, which could lengthen segments and enhance the mechanical properties more than the grafting reaction.

### 3.3. Optimization of Blends with DCP–GMA 1:2

#### 3.3.1. Rheological Properties

The longer the residence time in the extruder, the higher the complex viscosities exhibited in DCP–GMA 1:2 blends. Higher values point out a compatible interconnected molecular structure, which corresponds with the grafting reaction that promotes growing side chains, which strengthen interfacial interactions, along with the cross-linked network structure that restricts chain movement, leading to greater resistance to deformation under shear stress.

**Figure 22:**
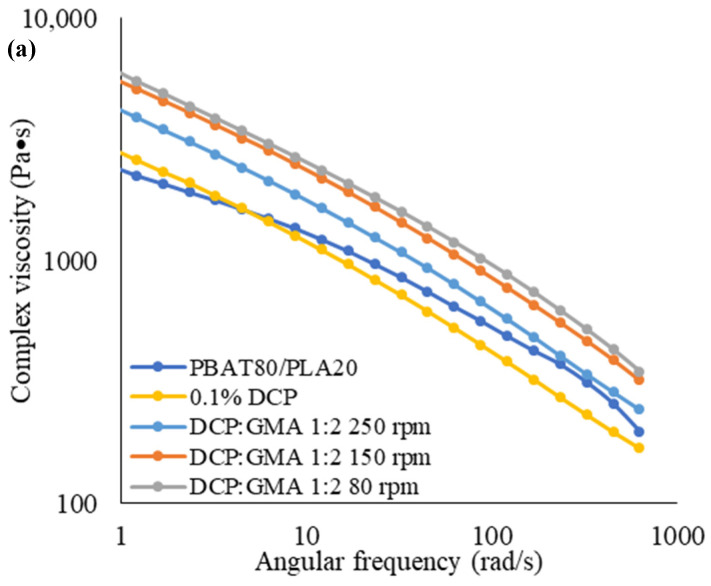

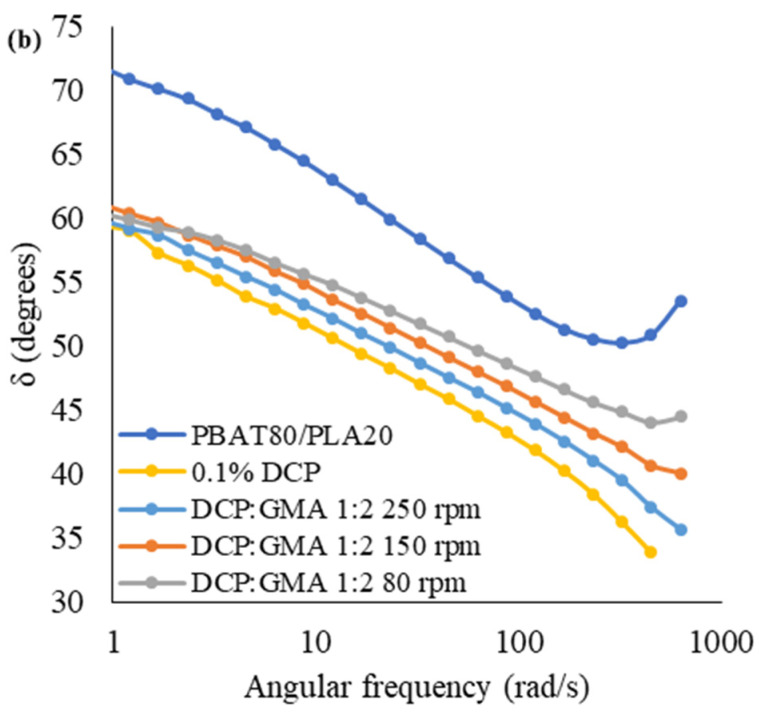
Rheological behavior of DCP–GMA 1:2 blends extruded at different screw speeds: (**a**) complex viscosity, (**b**) loss angle.

As seen in Figure 22b, lower screw speeds raised the phase angles in the tested frequency range, indicating a viscous behavior with significant energy dissipation.

**Figure 23:**
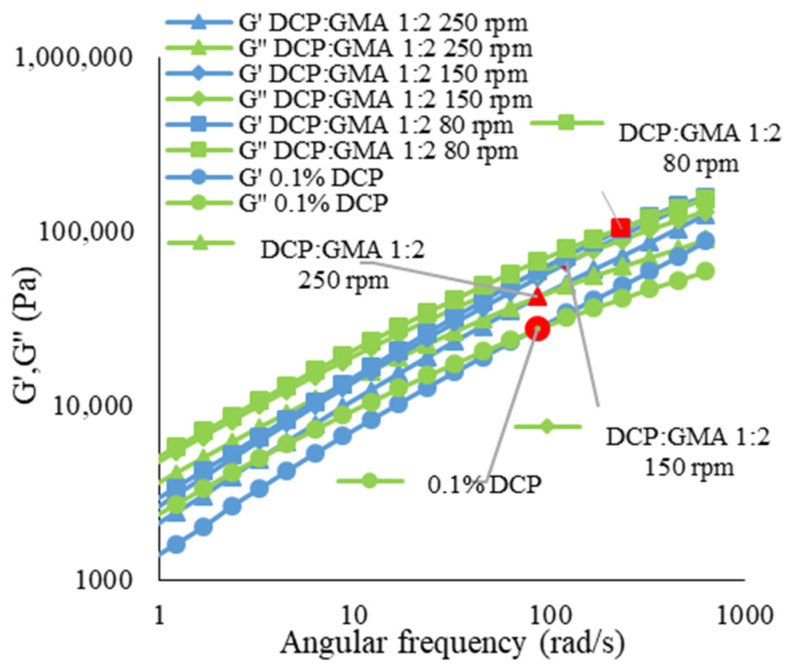
Storage and loss modulus crossover points from frequency sweep curves of DCP–GMA 1:2 blends extruded at different screw speeds.

Longer residence time in the extruder leads to scission polymer chains, especially targeting larger chains, thus reducing the average Mw yet narrowing MWD, exactly as seen in Figure 23 and Table S5 (increase in the modulus axis of the intersected G′, G″(ω) point in blends extruded at low screw speeds). Velghe et al. [56] deduced a supporting conclusion that although a longer residence time propagates shearing of the material, the cleaved chains can be reconnected by chain extenders and branching agents to enhance melt stabilization.

#### 3.3.2. Tensile Properties

**Figure 24:**
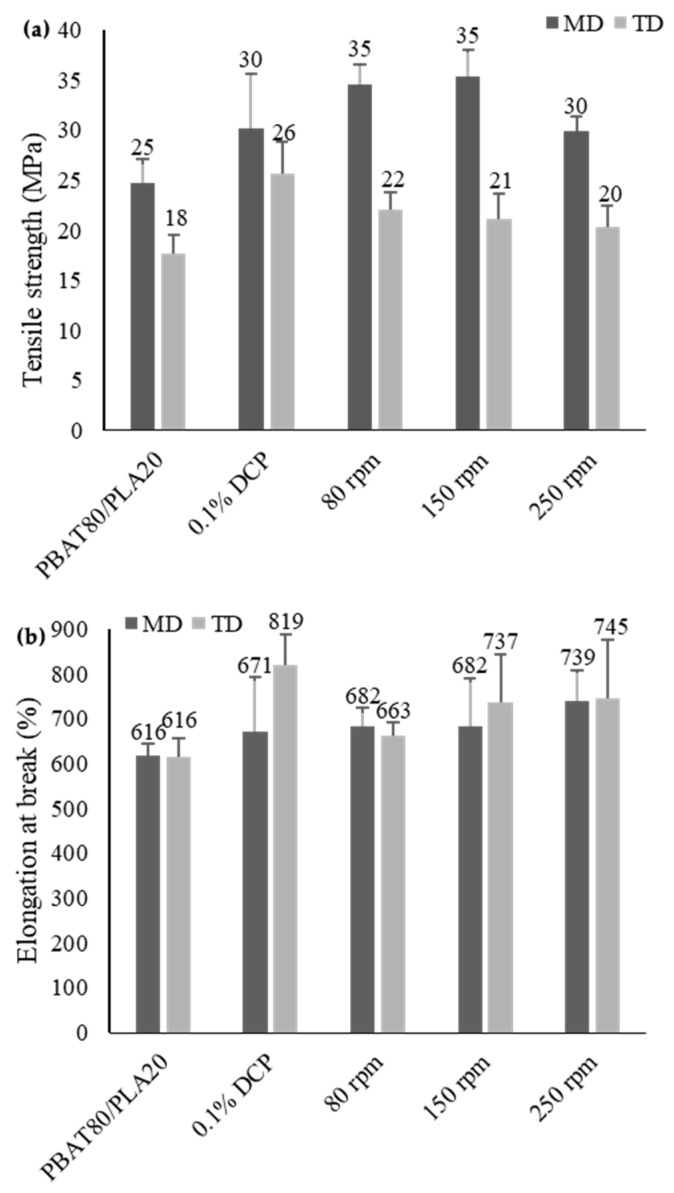
Tensile properties of DCP–GMA 1:2 blends extruded at different screw speeds: (**a**) tensile strength, (**b**) elongation at break.

According to Figure 24, lower screw speeds, i.e., longer residence time, increased tensile strength, and decreased elongation at break in both directions, and tensile strength of a blend that was extruded at 150 rpm exhibited improvement of 17% compared to the blend with 0.1% DCP (35 MPa compared to 30 MPa). A similar observation was made by Barbosa et al. [52] where higher feed rates, i.e., shorter residence time, decreased the elastic modulus in reactive extruded PLA70/PBAT30 with MA and DCP, indicating poorer interfacial adhesion and less homogeneous morphology between the PLA and PBAT phases due to shorter time for the radical and grafting reactions to occur.

The SEM images (Figure S3) demonstrate a finer-dispersed homogenous morphology and smoother surface fracture for the 80 rpm blend compared to the 250 rpm blend, reinforcing the argument that longer residence time in the extruder improves compatibilization, which is manifested in a smaller spherical dispersed phase due to the reduction in interfacial tension, and supporting the evidence of the increased tensile strength and the enhancement in complex viscosity values, as was discussed above.

The optimization demonstrated the need for a longer residence time/longer screw length to perform an effective reactive extrusion, in which the ring-opening reaction of the chain extenders would occur fully.

## 4. Conclusions

Reactive extrusion was carried out by crosslinking and grafting reactions to increase stretchability and melt strength in bio-degradable films. Chain extenders in the presence of peroxide were grafted onto the PBAT/PLA blend during batch mixing and compounding to produce cast-extruded films.

DCP addition promoted better dispersion and distribution of the PLA phase in the PBAT matrix due to improved interfacial adhesion, which resulted in elevated Mw, shear viscosity, and melt strength, as was seen in the rheological study. Its incorporation granted improved resistance to breakage under load, with a greater extent of deformation before rupture, indicating higher stretchability. The inward shift of PLA’s and PBAT’s T_g_ was evidenced by enhanced compatibility due to the crosslinking reaction. DMA and tensile tests affirmed that 0.1% DCP is the optimal radical initiator concentration in the studied blend.

In situ compatibilization was performed when MA, GMA, and DCP were blended in a batch mixer, yielding superior rheological properties—a dramatic rise in melt strength from 4838 Pa to 6940 Pa was displayed in the DCP–GMA 1:1 blend compared to the 0.1% DCP blend at a shear rate of 1 s^−1^. Enhanced miscibility was confirmed for the extruded blend with DCP–GMA 1:1, which displayed an inward shift of 0.5 °C in T_g_ compared to the blend with 0.1% DCP.

An investigation regarding the change in the processing method from batch mixer to extrusion was conducted thoroughly. Batch-mixed blends displayed greater viscosity improvement compared to extruded blends—shear viscosity of the DCP–GMA 1:1 batch-mixed blend was higher than the extruded blend by 38%. It was found that sufficient residence time is required for an effective reaction, which consists of a ring-opening reaction. Optimization of the residence time for the DCP–GMA 1:2 blend yielded an improvement of 17% in tensile strength compared to the blend with 0.1% DCP and led to higher complex viscosity values and a finer dispersed morphology.

## Figures and Tables

**Table 1:** Chain extender compositions (in molar ratio).

GMA	MA
0.25	0.25
0.5	0.5
1	1
2.5	2.5
5	5
0.5	0
1	0
2	0
5	0
10	0
0	0.5
0	1
0	2
0	5
0	10

**Table 2:** Chosen compositions.

DCP	GMA	MA
1	1	1
1	1	0
1	2	0
1	0	2

**Table 3:** Comparison between G′, G″(ω) batch-mixed and extruded crossover points.

	Batch-Mixed		Extruded	
Sample	Angular Frequency (rad/s)	Modulus (kPa)	Angular Frequency (rad/s)	Modulus (kPa)
PBAT80/PLA20	325.4	91.8	-	-
0.05% DCP	168.6	120.9	234.2	66.0
0.1% DCP	121.3	63.7	87.3	27.7
0.25% DCP	23.4	64.1	23.4	18.8
0.5% DCP	4.5	21.5	6.3	7.1

**Table 4:** Slope–intercept equations of the samples from Figure 7.

Sample	Slope	Intercept
PBAT80/PLA20	1.2558	−1.3386
0.05% DCP	1.2366	−1.1534
0.1% DCP	1.2165	−0.8206
0.25% DCP	1.2361	−1.0038
0.5% DCP	1.2636	−1.1472

**Table 5:** Thermal parameters of the second heating cycle for PBAT/PLA (80/20) blends with different DCP content: (1) first cooling cycle, (2) second heating cycle.

	T_g2_ PBAT (°C)	T_g2_ PLA (°C)	T_m2_ PBAT (°C)	ΔH_m2_ PBAT (J/g)	T_m2_ PLA (°C)	ΔH_m2_ PLA (J/g)	ΔH_cc1_ (J/g)	X_c2_ of PLA (%)	X_c2_ of PBAT (%)	T_c1_ (°C)	ΔH_c1_ (J/g)
PLA	-	57.8	-	-	153.2	5.1	4.9	0.2	-	95.4	0.4
PBAT	−27.5	-	121.9	13.6	-	-	-	-	11.9	30.3	20.1
PBAT80/PLA20	−30.6	59.7	121.3	8.4	-	-	-	-	9.2	66.3	14.6
0.05% DCP	−30.2	59.2	120.4	11.1	-	-	-	-	12.2	73.8	16.8
0.1% DCP	−31.3	58.8	120.9	9.4	151.1	0.8	-	-	10.3	77.0	16.0
0.25% DCP	−31.2	57.6	122.4	5.5	148.0	2.7	-	-	6.0	80.4	15.6
0.5% DCP	−30.8	59.1	123.0	3.3	146.6	2.3	-	-	3.6	81.8	14.8

**Table 6:** Pore size information.

Sample	Mean Pore Size (nm)	STDEV.S (nm)
PBAT80/PLA20	611.74	286.54
0.05% DCP	512.04	131.74
0.1% DCP	420.1	194.32
0.25% DCP	732.33	374.38
0.5% DCP	275.7	63.16

**Table 7:** Slope–intercept equations of the samples from Figure 18.

Sample	Slope	Intercept
PBAT80/PLA20	1.2558	−1.3386
0.1% DCP	1.2636	−1.1472
DCP–GMA–MA 1:1:1	1.2238	−1.0884
DCP–GMA 1:2	1.2747	1.1059−
DCP–MA 1:2	1.2171	1.1213−
DCP–GMA 1:1	1.2114	1.0867−

**Table 8:** Thermal parameters of PBAT/PLA (80/20) extruded blends with different GMA, MA, and DCP content: (1) first cooling cycle, (2) second heating cycle.

	T_g2_ PBAT (°C)	T_g2_ PLA (°C)	T_m2_ PBAT (°C)	ΔH_m2_ PBAT (J/g)	T_m2_ PLA (°C)	ΔH_m2_ PLA (J/g)	X_c2_ of PLA (%)	X_c2_ of PBAT (%)	T_c1_ (°C)	ΔH_c1_ (J/g)
DCP–MA 1:2	−30.0	59.3	121.8	6.7	-	-	-	9.2	74.1	13.9
DCP–GMA 1:2	−30.2	58.2	121.9	6.9	-	-	-	9.4	78.2	12.1
DCP–GMA 1:1	−31.9	58.9	122.9	6.8	-	-	-	9.3	76.7	13.1
DCP–GMA–MA 1:1:1	−29.3	61.1	123.8	5.9	-	-	-	8.0	74.6	13.7

**Table 9:** Pore size information.

Sample	Mean Pore Size (nm)	STDEV.S (nm)
DCP–GMA 1:1	1383.32	749.13
DCP–GMA–MA 1:1:1	1931.48	1160.79
DCP–GMA 1:2	1302.35	492.30
DCP–MA 1:2	780.98	390.37

## Data Availability

Data are contained within the article and Supplementary Materials.

## Supplementary Materials

The following supporting information can be downloaded at: https://www.mdpi.com/article/10.3390/molecules30153211/s1, Additional figures and tables from DMA, DSC, SEM, rheological, and tensile testing are available in the Supplementary Information. Figure S1: Calculated shift in Tg’s of the PLA and PBAT phase from the second heating cycle from Table 5; Figure S2: Calculated shift in Tg’s of the PLA and PBAT phase from the second heating cycle from Table 8; Figure S3: Morphology of DCP:GMA 1:2 blends extruded in different screw speeds: (a) 80 rpm, (b) 250 rpm; obtained by SEM, ×2500; Table S1: Tensile properties of PBAT/PLA (80/20) extruded blends with DCP; Table S2: DMA testing results for Figure 11b; Table S3: G’, G’’(ω) crossover points for Figure 15; Table S4: Tensile properties of PBAT/PLA (80/20) extruded blends with DCP, GMA, and MA; Table S5: G’,G’’(ω) crossover points for Figure 22; Table S6: Tensile properties of DCP:GMA 1:2 blends extruded in different screw speeds.

## Symbols and Abbreviations

Symbols and Abbreviations	Definitions
LDPE	Low-density Polyethylene
PP	Polypropylene
PBAT	Poly(butylene adipate-co-terephthalate)
PLA	Poly(lactic acid)
FIBC	Flexible intermediate bulk container
SEM	Scanning Electron Microscopy
DMA	Dynamical Mechanical Analysis
DSC	Differential Scanning Calorimetry
T_g_	Glass transition temperature
T_c_	Melt crystallization temperature
T_cc_	Cold crystallization temperature
T_m_	Melting temperature
ΔH_m_	Melt enthalpy
ΔH_c_	Crystallization enthalpy
X_c_	Degree of crystallinity
G′	Storage modulus
G″	Loss modulus
M_w_	Molecular weight
MWD	Molecular weight Distribution
ω	Angular frequency
δ	Loss angle
wt	Weight
η	Shear viscosity
L/D	Length/Diameter
ASTM	American Society for Testing and Materials
g	Grafted
STDEV.S	Standard deviation of a sample of the population
MD	Machine Direction
TD	Transverse Direction
S.I.	Supplementary Information
