# Supplementary material for: Stretchability and Melt Strength Enhancement of Biodegradable Polymer Blends for Packaging Solutions
Source: Molecules. 2025 Jul 31;30(15):3211. doi: 10.3390/molecules30153211 (PMC12348934; doi:10.3390/molecules30153211)
Supplement: Supplementary file 1 [file molecules-30-03211-s001.zip › molecules-3745312-supplementary.pdf]

Supplementary Information

Stretchability and melt strength enhancement of bio-degradable polymer blends for packaging solutions

Katy D. Laevsky, Achiad Zilberfarb, Ana L. Dotan, Amos Ophir

Department of Polymer Materials Engineering, Shenkar College of Engineering, Design and Art, Ramat-Gan, Israel.

***Corresponding author:** Ana L. Dotan, Department of Polymer Materials Engineering, Shenkar College of Engineering, Design and Art, Ramat-Gan 6262528, Israel. Email: adotan@shenkar.ac.il, ORCID: 0000-0001-6750-4164.

Molecules

1. Determination of optimal DCP content

1.1. Thermal analysis

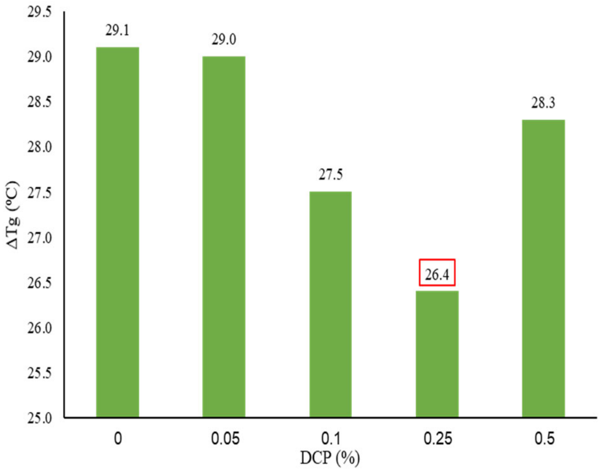

Figure S1 – Calculated shift in Tg's of the PLA and PBAT phase from the second heating cycle from Tab. 5

1.2. Tensile properties

Table S1 – Tensile properties of PBAT/PLA (80/20) extruded blends with DCP

Samples	σ (MPa)		ε_b (%)	
	**MD**	**TD**	**MD**	**TD**
PBAT80/PLA20	25 ± 2	18 ± 2	616 ± 27	616 ± 41
0.05% DCP	25 ± 4	21 ± 2	585 ± 93	734 ± 75
0.1% DCP	30 ± 5	26 ± 3	671 ± 121	819 ± 68
0.25% DCP	25 ± 3	23 ± 2	637 ± 51	775 ± 54
0.5% DCP	22 ± 2	18 ± 2	636 ± 53	705 ± 58

1.3. Dynamical-mechanical analysis

Table S2 – DMA testing results for Fig. 11(b)

Sample	Tg PBAT (^{o}C)	Tg PLA (^{o}C)
PBAT80/PLA20	-22.1	64.4
0.05% DCP	-25.1	62.8
0.1% DCP	-21.5	71.6
0.25% DCP	-22.7	65.8
0.5% DCP	-22.8	63.5

2. Incorporation of chain extenders

2.1. Rheological properties

Table S3 – G', G''(ω) crossover points for Fig.15

Sample	Angular Frequency (rad/s)	Modulus (kPa)
0.1% DCP	87.3	27.7
DCP:GMA 1:1	168.56	119.1
DCP:GMA 1:2	32.5	12.3
DCP:MA 1:2	325.4	130.6
DCP:GMA:MA 1:1:1	121.3	70.6

2.2. Thermal analysis

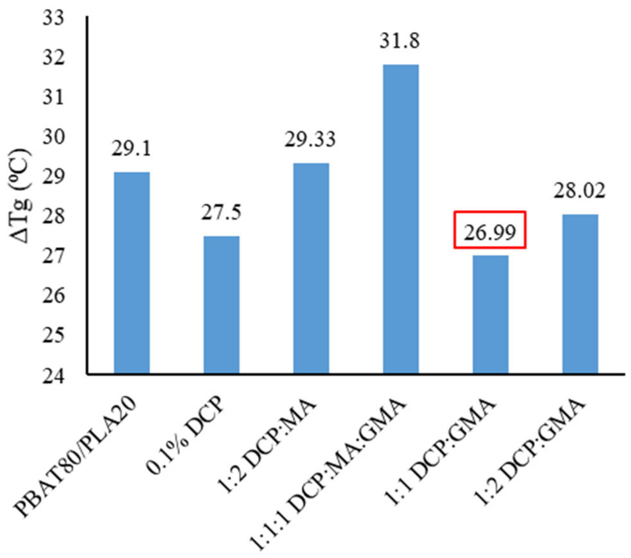

Figure S2 – Calculated shift in Tg's *of the PLA and PBAT phase from the second heating cycle from Tab. 8*

2.3. Tensile properties

Table S4 – Tensile properties of PBAT/PLA (80/20) extruded blends with DCP, GMA, and MA

Samples	σ (MPa)		ε_b (%)	
	**MD**	**TD**	**MD**	**TD**
DCP:GMA 1:1	27 ± 3	20 ± 2	643 ± 50	656 ± 35
DCP:GMA 1:2	27 ± 3	21 ± 1	637 ± 59	658 ± 47
DCP:MA 1:2	28 ± 2	22 ± 1	686 ± 34	743 ± 33
DCP:GMA:MA 1:1:1	28 ± 1	22 ± 2	691 ± 23	709 ± 34

3. Optimization of blends with DCP:GMA 1:2

3.1. Rheological properties

Table S5 – G',G"(ω) crossover points for Fig.22

Sample	Angular Frequency (rad/s)	Modulus (kPa)
0.1% DCP	87.3	27.7
DCP:GMA 1:2 80 rpm	234.2	104.5
DCP:GMA 1:2 150 rpm	121.3	67.3
DCP:GMA 1:2 250 rpm	87.3	42.0

3.2. Tensile properties

Table S6 – Tensile properties of DCP:GMA 1:2 blends extruded in different screw speeds

Samples	σ (MPa)		ε_b (%)	
	MD	TD	MD	TD
DCP:GMA 1:2 80 rpm	35 ± 2	22 ± 2	682 ± 43	663 ± 28
DCP:GMA 1:2 150 rpm	35 ± 3	21 ± 2	682 ± 107	737 ± 107
DCP:GMA 1:2 250 rpm	30 ± 1	20 ± 2	739 ± 70	745 ± 132

3.3. Morphological properties

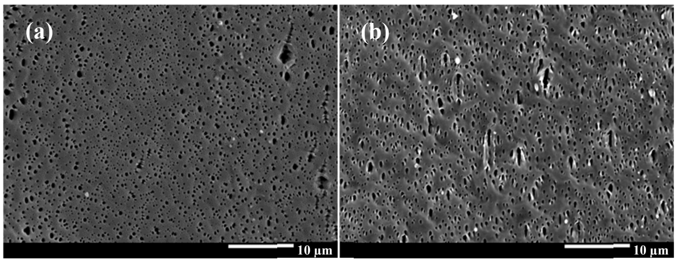

Figure S3 – Morphology of DCP:GMA 1:2 blends extruded in different screw speeds: (a) 80 rpm, (b) 250 rpm; obtained by SEM, ×2500.
